# Membranoproliferative glomerulonephritis complicating Waldenström’s macroglobulinemia

**DOI:** 10.1186/1471-2369-13-172

**Published:** 2012-12-21

**Authors:** David Kratochvil, Kerstin Amann, Heike Bruck, Maike Büttner

**Affiliations:** 1 Department of Nephrology, University Hospital Essen, University of Duisburg-Essen, Hufelandstr. 55, 45122, Essen, Germany; 2 Institute of Pathology, Department of Nephropathology, University Hospital Erlangen, Krankenhausstr. 8-10, 91054, Erlangen, Germany

**Keywords:** Waldenström’s macroglobulinemia, MPGN, Hyaline thrombi

## Abstract

**Background:**

Lymphoproliferative disorders causing paraproteinemia can be associated with various kidney injuries including the deposition of monoclonal immunoglobulins (Ig). A known glomerular manifestation of Waldenström’s macroglobulinemia is characterized by prominent intracapillary hyaline thrombi and lack of conspicuous glomerular proliferation. The present case was special in 2 aspects: 1. the diagnosis of glomerulonephritis was unexpected before renal biopsy, 2. the prominent glomerular proliferation paired with large intracapillary hyaline thrombi is uncommon in Waldenström’s macroglobulinemia-associated glomerulonephritis.

**Case presentation:**

A 73-year-old Caucasian woman with a long-standing history of rheumatoid arthritis and Waldenström’s macroglobulinemia was admitted for acute renal failure (ARF), which initially was presumed to be the consequence of extrarenal causes. Proteinuria and hematuria were only mild. In renal core biopsy, a membranoproliferative glomerulonephritis (MPGN) and prominent intracapillary hyaline monoclonal IgM thrombi were found in addition to acute tubular necrosis. Of note, the patient’s history was positive for purpuric skin changes, suspicious for cryoglobulinemia. However, serological tests for cryoglobulins were repeatedly negative. The ARF resolved before the start of immunomodulatory therapy for Waldenström’s macroglobulinemia.

**Conclusion:**

The presence of MPGN with prominent hyaline thrombi in the context of Waldenström’s macroglobulinemia is uncommon and can be oligosymptomatic. We discuss this case in the context of previous literature and classifications suggested for monoclonal Ig-related renal pathologies.

## Background

Lymphoproliferative malignancies producing monoclonal immunoglobulins may cause different nephropathies. Compared to multiple myeloma, renal involvement in Waldenström’s macroglobulinemia (WM) is much more seldom and often manifests as IgM deposits along the glomerular basement membranes, amyloidosis or lymphomatous infiltration of the renal parenchyma, whereas cast nephropathy is almost never observed [1-4]. Morel-Maroger et al. described a renal manifestation of WM characterized by prominent intracapillary IgM thrombi and lack of relevant glomerular proliferation [3]. This disease pattern has also been referred to as intracapillary monoclonal deposits disease (ICMDD) in the context of any IgM-secreting monoclonal proliferation [1]. Prominent intracapillary hyaline thrombi can also develop in the context of cryoglobulinemia, containing a monoclonal immunoglobulin (Ig) often in the context of lymphoproliferative disorders (type I) or polyclonal with or without monoclonal Ig (type II and III, respectively), which may be associated with persistent infections and autoimmune syndromes [5,6]. Although about 15% of WM cases are associated with type I cryoglobulins (CG), few patients have symptoms or complications [2]. Cryoglobulinemic glomerulonephritis (CG-GN), often reported in type II cryoglobulinemia, is an exceedingly rare finding in WM [7].

## Case presentation

**Figure 1:**
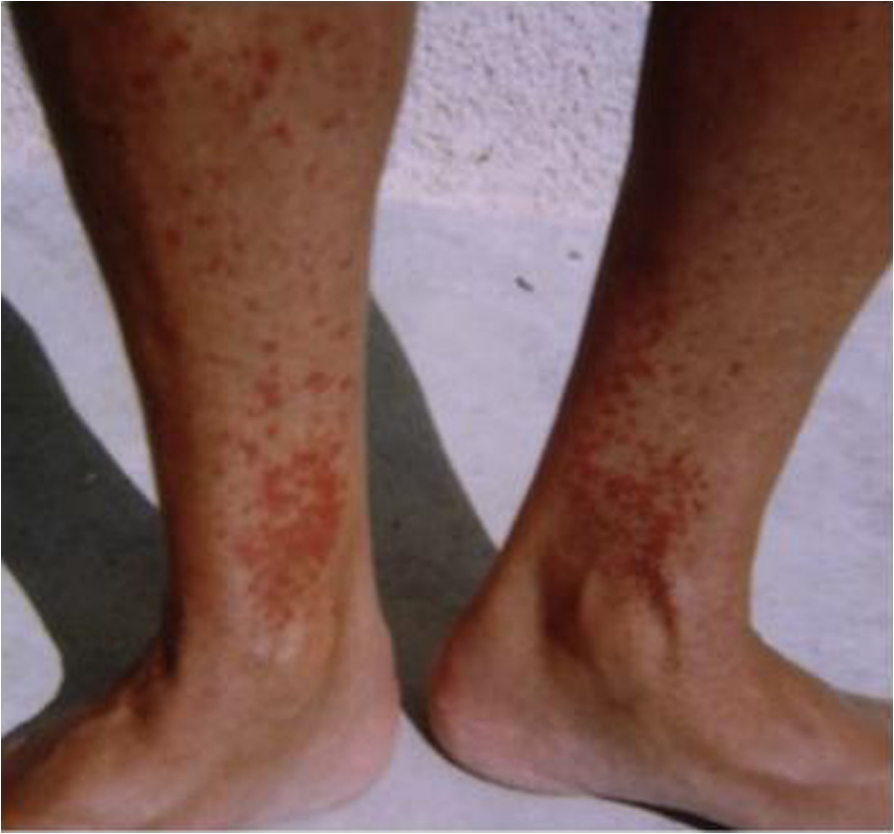
**Purpuric skin changes of the lower legs.** Photograph taken 7 years prior to admission and five years after the initial diagnosis of RA showing purpura of the legs.

A 73-year-old Caucasian woman was transferred to the renal department for acute renal failure (ARF) thought to be primarily due to extrarenal causes. Her medical history was notable for rheumatoid arthritis (RA) diagnosed 12 years ago, accompanied by purpuric skin lesions on the extensor surfaces of her lower legs (Figure 1, a picture taken 7 years ago) and arms. Five months ago, one lesion developed into a pretibial ulcer on her right leg, for which she underwent skin grafting. 5 years ago, serum protein studies revealed a monoclonal IgM gammopathy. A bone marrow biopsy performed 4 months ago because of worsening anemia yielded the diagnosis of lymphoplasmacytic lymphoma. Her recent medical history was marked by progressive dyspnea due to pleural effusions. Before transfer to our hospital, the serum creatinine was 2.4 mg/dl, as compared to 1.07 mg/dl five months ago.

At physical examination, the patient’s temperature was 38.2°C and her blood pressure was 110/70 mmHg. At the lung bases, the chest was dull to percussion with diminished breath sounds. The cardiac and abdominal examinations were normal. The extremities were remarkable for pitting ankle edema. The hands showed ulnar deviation and swan neck deformity. The skin revealed an unremarkable skin grafting wound on her right, and discrete palpable purpura on her left pretibial region. Renal ultrasound was normal. Although cardiac decompensation was suspected because of dyspnea, edema and pleural effusions and an elevation of BNP (2108 pg/ml), echocardiography showed only a slightly decreased cardiac ejection fraction (40-50%).

Laboratory data included a serum creatinine of 2.54 mg/dl and BUN of 96 mg/dl. Urine dipstick evaluation showed 2+ blood and no protein. The urinary sediment contained 292 erythrocytes/μl without dysmorphic forms. Additional laboratory tests performed during hospitalization included: high levels of rheumatoid factor (881 IU/ml) and anti-CCP antibodies (>340 U/ml), normal ANA serology, and negative hepatitis C serology. Urinary protein excretion was 1.23 g/24 h, Bence-Jones lambda was 226 mg/dl and Bence-Jones kappa 77.2 mg/dl. The serum protein electrophoresis showed a monoclonal IgM lambda band; serum IgM was 31.4 g/l. Despite repeated testing, CG serology remained negative.

A renal biopsy was performed.

**Figure 2:**
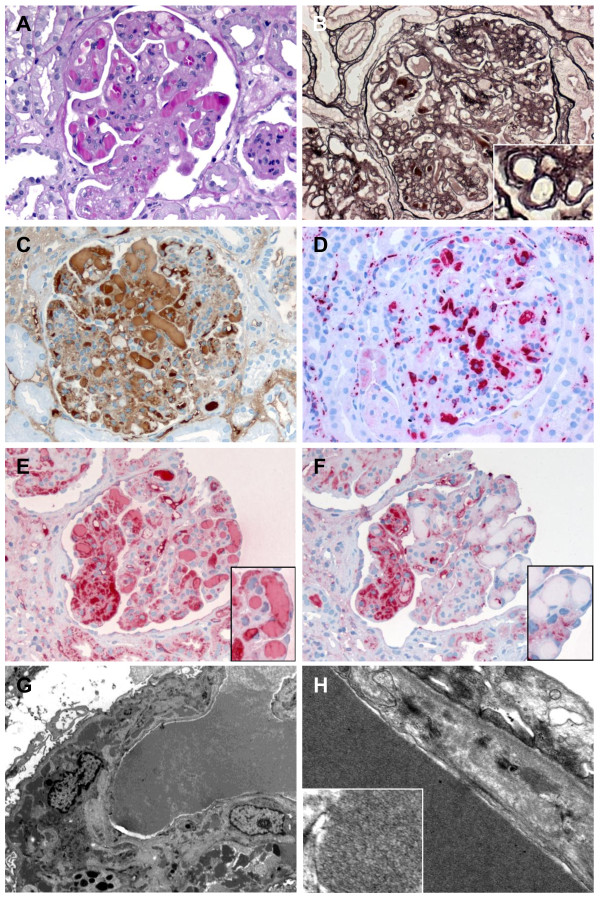
**Light microscopy, immunohistochemistry and electron microscopy of MPGN with intracapillary IgM thrombi with lambda light-chain restriction. (A)** Periodic-acid Schiff (PAS) staining (400x) with numerous intracapillary PAS positive thrombi and prominent intracapillary and mesangial proliferation, **(B)** Silver staining with double contoured basement membranes as highlighted in the inlay (400x), **(C)** IgM immunohistochemistry with strong staining of the thrombi and basement membranes (400x). **(D)** CD68 immunohistochemistry showing dense mesangial and intracapillary infiltration of the glomeruli by monocytes/macrophages (400x). **(E)** lambda (400x) and **(F)** kappa (400x) light-chain immunohistochemistry with a lambda light-chain restriction in the thrombi. Inlays show thrombi (630x). **(G)** Electron microscopy showing a basement membrane with segmental cellular interposition and subendothelial and intramembranous electron-dense deposits as well as an intracapillary thrombus (3597x). **(H)** The electron-dense deposits show a granular structure. No tubulo-reticular structures are present (21560x, inlay 60000x). Magnifications are indicated as original magnifications.

Light microscopy showed renal parenchyma with 31 glomeruli (one obliterated), which were hypercellular with prominent mesangial and intracapillary proliferation, prominent intracapillary PAS-positive thrombi in most glomeruli and double contoured basement membranes (Figure 2A and B). Extracapillary proliferation was absent. In immunohistochemical stainings, the thrombi were strongly reactive for IgM and lambda light chains without reactivity for kappa light chains, IgA, IgG, C1q and C3c. A granular peripheral positivity of the glomerular capillaries was seen in IgM, IgG and C3c as well as in the lambda and to a lesser extent kappa light chain stainings. CD68 staining showed a dense histiocytic infiltration of the glomeruli (Figure 2C-F). Congo red staining was negative. Signs of acute tubular necrosis were present.

In electron microscopy (EM) a prominent increase in mesangial matrix and mesangial and intracapillary hypercellularity with numerous inflammatory cells were seen (Figure 2G and H). The glomerular basement membranes focally showed mesangial cell interposition. Podocytes presented with foot-process effacement. The most striking finding was intracapillary deposits of electron-dense granular material, which, even at very high magnification, showed no fingerprint or tubuloreticular structures. Electron-dense deposits with identical structure were found subendothelially and intramembranously.

The lesion was classified as membranoproliferative glomerulonephritis (MPGN) with intracapillary IgM deposits in the context of WM accompanied by acute tubular necrosis. Because of the pattern of glomerulonephritis with an MPGN-like picture and prominent hyaline thrombi, an association with cryoglobulinemia was suggested.

In the further clinical course the ARF subsided without specific treatment and creatinine levels declined from 3.04 mg/dl to 1.3 mg/dl three weeks after admission. Nevertheless, we initiated an immunomodulatory therapy with rituximab, after which renal function continued to fluctuate. Repeated CG testing remained negative.

## Conclusions

The reported case of MPGN with intracapillary thrombi is remarkable in several aspects.

First, because of heart failure signs and highly elevated BNP level, we suspected that ARF was due to cardiac decompensation as an extrarenal cause. The only slightly abnormal echocardiographic findings could be explained by the fact that at the time of the examination, cardiac function was already recovering from an acute event. The urine analysis showed only mild proteinuria and hematuria without dysmorphic erythrocytes, so that no distinct glomerular disease was expected. However, renal biopsy, in addition to the expected acute tubular necrosis, revealed a proliferative glomerulonephritis necessitating a specific immunomodulatory therapy. The renal function recovered before the start of the therapy, again arguing for a prerenal cause of acute renal failure. Thus, the finding of the glomerulonephritis was ‘incidental’ in the context of a renal biopsy performed for acute renal failure. Moreover, proliferative GN reported in the context of monoclonal lymphoproliferations is often associated with nephrotic syndrome [1,8,9], which was not true for our case, so that the MPGN pattern of injury was even less expected. Secondly, from the morphological point of view our case was reminiscent of CG-GN with monoclonal type I CG [10,11], which would go very well in line with the glomerular proliferation and the purpuric skin manifestation. While a microtubular or fingerprint ultrastructure on EM is very specific for CG-GN, a finding of granular deposits as seen in our case does not prove or exclude this disorder [10,12]. Nevertheless, CG-GN could not be proven because of repeatedly negative CG testing.

Another entity that might be considered in the differential diagnosis is WM-associated nephropathy with prominent intracapillary thrombi and, contrary to our case, lack of relevant proliferation or histiocytic infiltration [1,3].

In monoclonal deposit nephropathies, CG presence very often is seen tied to the feature of cellular proliferation [10]. However, several reports in literature can be found describing monoclonal intrarenal IgM deposits and marked glomerular proliferation sometimes with the typical picture of MPGN *without* proof of CG [1,4,8,13-15]. Interestingly, negativity for CG is also present in a recently described proliferative glomerulonephritis with monoclonal IgG deposits (PGNMID) [16]. Comparable to this entity associated with IgG, recently a case of proliferative GN with monoclonal IgM deposits, but without association with WM, was reported [9]. This showed several similarities with the present case, including lack of evidence of CG, presence of a monoclonal IgM and prominent proliferation [9], so that this case and ours might both be classified as proliferative GN with monoclonal IgM deposits. In contrast to other reported cases that might belong to this group [9] our patient showed a lambda light chain restriction and was female, so that apparently this pattern of injury is not restricted to men and kappa light chain producing lymphoproliferation. The concomitantly observed minor peripheral capillary deposits of IgG, kappa light chains and C3c might either represent a trapping of immunoglobulins and complement in the widened basement membranes or be due to an immune activation maybe caused by the monoclonal IgM.

Presence of CG, conversely, is not always accompanied by prominent glomerular proliferation [3,17]. Thus, glomerulopathies associated with monoclonal gammopathies include a spectrum of proliferative and non-proliferative GN which can, but need not be associated with the presence of CG. In addition, RA and its treatments can also lead to MPGN [18]. Therefore it cannot be excluded that in the present case RA might be involved in the pathogenesis of MPGN.

Treatment for WM is usually indicated for conditions like progressive, severe anemia and hyperviscosity syndrome [19], for which there was no evidence in the present patient. Given the impressive renal changes linked to deposition of monoclonal antibodies, we nevertheless felt the urge to treat and suppress their production. The optimal treatment regimen for intrarenal monoclonal deposit disorders has yet to be defined. In general, treatment of the malignancy is expected to alleviate glomerulopathy [13]. Because of the patient’s poor general condition, we opted for monotherapy with rituximab. This case illustrates the need for renal biopsy in ARF patients with a positive history for diseases that may be associated with more unusual forms of nephropathy.

## Consent

Written informed consent was obtained from the patient’s husband for the publication of this Case report. A copy of the written consent is available for review by the Series Editor of this journal.

## Abbreviations

ARF: Acute renal failure; CG: Cryoglobulins; CG-GN: Cryoglobulinemic glomerulonephritis; EM: Electron microscopy; ICMDD: Intracapillary monoclonal deposits disease; Ig: Immunoglobulin; MPGN: Membranoproliferative glomerulonephritis; PGNMID: Proliferative GN with monoclonal IgG deposits; RA: Rheumatoid arthritis; WM: Waldenström’s macroglobulinemia.

## Competing interests

The authors declare that they have no competing interests.

## Authors’ contributions

D.K. has made substantial contributions to the design and conception of the discussion, he has collected the clinical data, was involved in the interpretation of the data and writing of the manuscript. K.A. has participated in making the histomorphological and electron microscopical diagnosis, has been involved in the drafting and revision of the manuscript and was involved in the discussion of critical intellectual content. H.B. was involved in the interpretation of the clinical data and the drafting and revision of the manuscript and was involved in the discussion of critical intellectual content. M.B. has made substantial contributions to the design and conception of the manuscript, made the pathological diagnosis together with K.A. and substantially participated in the writing of the manuscript. All authors read and approved the final manuscript.

## Pre-publication history

The pre-publication history for this paper can be accessed here:

http://www.biomedcentral.com/1471-2369/13/172/prepub
